# The Serum Level of IL-1B Correlates with the Activity of Chronic Pulmonary Aspergillosis

**DOI:** 10.1155/2018/8740491

**Published:** 2018-09-27

**Authors:** Mengling Zhan, Benyong Xu, Lan Zhao, Bing Li, Liyun Xu, Qiuhong Sun, Jun Zhang, Zhemin Zhang, Haiqing Chu

**Affiliations:** ^1^Tongji University School of Medicine, Shanghai 200092, China; ^2^Department of Respiratory Medicine, Shanghai Pulmonary Hospital, Tongji University School of Medicine, Shanghai 200433, China; ^3^Division of Hematology, Oncology and Blood & Marrow Transplantation, Department of Internal Medicine, Holden Comprehensive Cancer Center, University of Iowa Carver College of Medicine, Iowa City, IA 52242, USA

## Abstract

**Background:**

Until now, there have been no objective criteria to determine the activity of chronic pulmonary aspergillosis (CPA). This study aims to analyze the correlation between serum level of IL-1B and the activity of CPA and to determine whether serum IL-1B could be used to assess the activity of CPA.

**Methods:**

A total of 469 newly diagnosed CPA patients were enrolled. Correlation analysis in the whole subjects showed that only IL-1B level was associated with the activity of CPA. Then, 381 cases with factors significantly affecting IL-1B expression was excluded through multiple linear regression; the remaining 88 patients were divided into high IL-1B group and low IL-1B group, according to the median value of serum IL-1B, for subgroup analysis. A retrospective comparative analysis was subsequently performed between the two groups, including the clinical manifestation, microbiology and laboratory tests results, and imaging findings. We further investigated the relationship between IL-1B levels and CT characteristic which acted as the indicator of CPA activity, as well as changes in IL-1B level before and after surgery.

**Results:**

For all patients, correlation analysis revealed that IL-1B level correlated with both cavitary diameter (*P*=0.035) and aspergilloma size (*P*<0.047) but not with the thickness of the cavity (*P*=0.479). In subgroup comparative analysis, CT characteristics suggested that high activity of CPA, such as cavitary (27/44 vs 13/44, *P*=0.003) and aspergilloma lesions (25/44 vs. 11/44, *P*<0.002), were more frequently found in high IL-1B group. The cavity diameter (*P*<0.001), aspergilloma size (*P*=0.006), and cavity wall thickness (*P*=0.023) were significantly different between the two groups. When Spearman correlation analysis was performed once again in subgroup, an even stronger relationship of serum IL-1B with the cavity diameter (*Rs*=0.501, *P*=0.002) and aspergilloma size (*Rs*=0.615, *P*=0.001) was observed. Interestingly, a significant reduction of IL-1B level was observed after successful resection of CPA lesions.

**Conclusion:**

Higher level of serum IL-1B is associated with more severe cavitary and aspergilloma lesions, which are indicative of more active CPA. In addition, IL-1B level reduced accordingly after lesion resection. Measuring IL-1B level therefore could be served as a convenient method to monitor the activity of CPA and be a potential predictive/prognostic marker for treatment response.

## 1. Introduction

Chronic pulmonary aspergillosis (CPA) is a chronic form of debilitating aspergillus infection that affects patients with pre-existing structural lung diseases [1]. It was reported that CPA affected 240,000 people in Europe and caused tremendous global burden following pulmonary tuberculosis (TB) [2–4]. CPA may also result in significant mortality, with a 5-year survival rate of 17.5%–62% without surgical resection and 15%–20% without any intervention [5–7].

The relationship between cytokines and fungal infections has been gaining interest among clinicians and scientists. Various studies on cytokine gene single-nucleotide polymorphisms (SNP), expression level, and related pathways in fungal infections have demonstrated that distinct cytokine profiles are closely related to inflammasome activation and disease activity, some of which suggested that cytokines may correlate with the activity of CPA [8–11]. For example, several papers reported that interleukin (IL)-1, IL-15, and interferon-*γ* could contribute to the pathogenesis in chronic cavitary pulmonary aspergillosis (CCPA) [8–10] and reduced CD40L and sCD40L, as well as increased IL-10 may compromise the immune response against aspergillus in patients with chronic necrotizing pulmonary aspergillosis (CNPA) [11].

IL-1 has been considered as a crucial cytokine for hosts to defend against a broad range of pathogens. The classical IL-1 cytokines are IL-1A and IL-1B [12]. Over the past years, the IL-1 cytokines, particular IL-1B, have been studied for their role in the antifungal host response against *Aspergillus fumigatus* infection. IL-1B is primarily produced by innate immune cells such as monocytes, macrophages, and dendritic cells upon activation. It is a potent proinflammatory cytokine that is tightly regulated by immune cells. Previous studies have demonstrated that active and growing fungi, such as swollen conidia, germinated conidia [13], hyphal fragments [14], and live conidia [15], were more potent in inducing IL-1B expression, and persistently elevated IL-1B could be a sign of strong fungal activity as well as on-going inflammation induced by host immune response to active fungus. However, evidence supporting a direct link of IL-1B with CPA is still lacking. Thus, we hypothesize that IL-1B also plays an import role in chronic pulmonary aspergillosis.

In the present study, we have endeavored to show that only serum IL-1B, among several proinflammatory cytokines and other traditional measures of inflammation, acted as a promising disease biomarker in predicting the activity of CPA, by exploring its association with the patients' clinical manifestations, microbiological manifestations, laboratory test results, and imaging findings.

## 2. Patients and Methods

### 2.1. Patient Selection

A total of 488 newly diagnosed CPA patients were recruited, among which, 19 subjects were excluded due to lack of complete image and blood inflammatory cytokine data. Thus, finally 469 subjects participated in the current study (Table 1). Patients were initially diagnosed and treated in Shanghai Pulmonary Hospital, Tongji University, from January 2009 to December 2015. The diagnostic criteria were in accordance with the European Society for Clinical Microbiology and Infectious Diseases (ESCMID) and the European Respiratory Society (ERS) Guideline of 2016 [16]. All the patients were documented well with detailed clinical and radiographic information. Microbiological and laboratory results of sputum, bronchial lavage fluid, and blood were collected before initial diagnosis. The multiple linear regression model for predicting serum IL-1B (using patient baseline characteristics, treatment status, and associated diseases as covariates in all the subjects with CPA) was conducted to screen for factors influencing serum IL-1B (Table 2). A total of 381 cases with any of these factors were excluded. The remaining 88 patients with CPA were divided into the high and low IL-1B groups according to the median serum IL-1B concentration of 20.3 ng/L for further subgroup analysis (Table 1). Among these patients, 22 received surgical treatment, and changes in serum IL-1B were evaluated. The study was approved by the Ethical Committee of Tongji University, Shanghai Pulmonary Hospital. All the participants signed informed consent for any procedures that are relevant to this study.

### 2.2. CT Scanning

All CT images were obtained from the Radiology Department of Shanghai Pulmonary Hospital. CT scanning was performed within 1 week of CPA diagnosis. Multislice spiral CT (Philips Brilliance 64) was used for routine CT scanning with 5 mm thick images at 5 mm intervals. High-resolution computed tomography (HRCT) images were obtained by scanning using 1 mm thickness at 10 mm intervals. The scanning range was from the lung apex to the costophrenic angle under the maximum inhalation. CT images were analyzed and interpreted by two radiologists and a pulmonologist who were blinded for the microbiological test results. All images were transmitted to the PACS system, and after multislice recombination, the three-dimensional image was reconstructed and then measured. The diameter of the Aspergilloma was measured in each layer, and direction and the maximum value were taken. The cavity diameter was defined as the average of the anteroposterior, transverse, and axial diameter measured in the mediastinal window. The thickness of the cavity wall was gauged in the front, rear, left, and right wall, and the average was taken. The largest lesion was measured when the lesion was multiple. The final interpretation was established by the consensus achieved among those three experts. The radiographic evaluation was based on Agarwal et al. and Godet et al.'s study [17, 18].

### 2.3. Blood Inflammatory Cytokines Test

All patients had fasted for more than 12 hours before 5 ml of peripheral venous blood was extracted in the next morning. The blood test was done around the time when the CT scan was performed. For the postoperative blood test, the blood samples were collected 3 months after surgery. All collected peripheral blood samples were centrifuged for 15 min (4°C; 3000 r/min), and the sera were collected for determination the level of inflammatory cytokines including IL-1B, IL-2, IL-6, IL-5, tumor necrosis factor- (TNF-) *α* and interferon-*γ*. Enzyme-linked immunosorbent assay (ELISA) was used, and the ELISA kit was purchased from BioLegend, Inc (US).

### 2.4. Statistical Analysis

All statistical analyses were conducted using SPSS20.0 (IBM, Armonk, NY, USA). The data were compared using Student's *t*-test (normally distributed data) or Mann–Whitney *U* test (nonnormally distributed data) for continuous variables, and Pearson *χ*^2^ test or Fisher's exact test for categorical variables. Student's paired *t*-test was performed to compare the pre- and postoperative serum IL-1B levels in selected CPA patients. Correlations were examined with Spearman's rank test. Serum IL-1B levels were analyzed by multiple stepwise regression until the most parsimonious model was achieved. Univariate *P* values of 0.2 entered the multivariate model. *P* values of <0.05 were considered statistically significant.

## 3. Results

### 3.1. Patient Characteristics

Eligible patients' characteristics, CT findings, and related test results for all patients and patients in subgroup are shown in Table 1. Based on the median value (20.3 ng/L, ranged from 0 to 499 ng/L), 44 cases were classed in high IL-1B group and 44 in low IL-1B group. There was no difference in basic characteristics, common laboratory test, aspergillus antibody, and GM test results between the two groups. Other commonly used cytokines such as IL-2, IL-6, IL-5, TNF-*α*, and interferon-*γ* revealed no significant difference as well (Table 1).

### 3.2. Relationship between Serum IL-1B Level and Radiographic Findings

**Figure 1:**
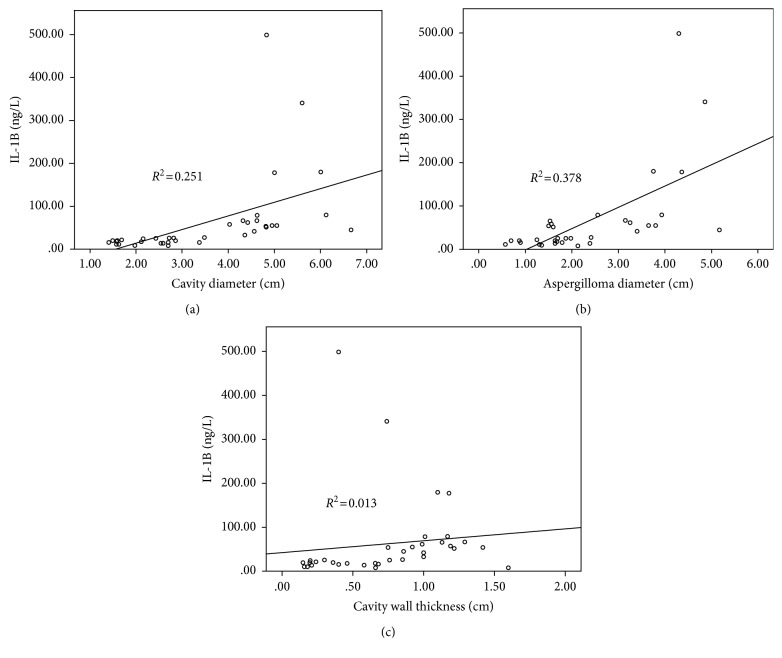
Trend analysis and correlation analysis found that IL-1B was correlated with the cavity diameter (*Rs*=0.501, *P*=0.002) (a) and aspergilloma size (*Rs*=0.615, *P*=0.001) (b), but not with the cavity wall thickness (*Rs*=0.114, *P*=0.496) (c).

For all patients, Spearman's rank correlation analysis revealed a mild but statistically significant correlation of IL-1B level with both cavitary diameter (*P*=0.035) and aspergilloma size (*P*=0.047), but not with the thickness of the cavity (*P*=0.479) (Table 3). In view of numerous accompanying factors and comorbidities potentially affecting the immunological status in our recruited subjects, we constructed a multiple linear regression model for predicting serum IL-1B using age, gender, BMI, smoking status, TB, NTM infection, lung abscess, other acute respiratory infection, cancer, COPD, AIP, sarcoidosis, other interstitial diseases, diabetes mellitus, hypertension, cardiac disease, cerebrovascular disease, systemic CS or IS therapy, and pulmonary surgery history as covariates in all the subjects with chronic pulmonary aspergillosis to screen for factors influencing serum IL-1B. The results indicated that active tuberculosis infection, NTM infection, lung abscess, other acute respiratory infections, cancer, AECOPD, AIP, and sarcoidosis positively correlated with serum IL-1B, while systemic CS or IS therapy led to a suppression in IL-1B expression. Accordingly, a total of 381 cases with any of these factors were excluded, and a subgroup analysis was performed in the remaining 88 subjects. When Spearman's correlation analysis was performed once again, and an even stronger relationship of serum IL-1B with the cavity diameter (*Rs*=0.501, *P*=0.002) and aspergilloma size (*Rs*=0.615, *P*=0.001) was observed (Figure 1). Besides, CT characteristics suggested that high activity of CPA, such as cavitary (27/44 vs 13/44, *P*=0.003) and aspergilloma lesions (25/44 vs. 11/44, *P*=0.002), were more frequently found in high IL-1B group. The cavity diameter (median, 4.6 cm (IQR, 2.8 to 5.0 cm) vs. 2.0 cm (IQR, 1.6 to 2.7 cm); *P*=0.001), aspergilloma size (median, 2.5 cm (IQR, 1.6 to 3.8 cm) vs. 1.5 cm (IQR, 0.9 to 1.9 cm); *P*=0.006), and cavity wall thickness (median, 0.9 cm (IQR, 0.4 to 1.2 cm) vs. 0.4 cm (IQR, 0.2 to 0.7 cm); *P*=0.023) were significantly different between the two groups.

### 3.3. Changes in Serum IL-1B before and after Surgery

**Figure 2:**
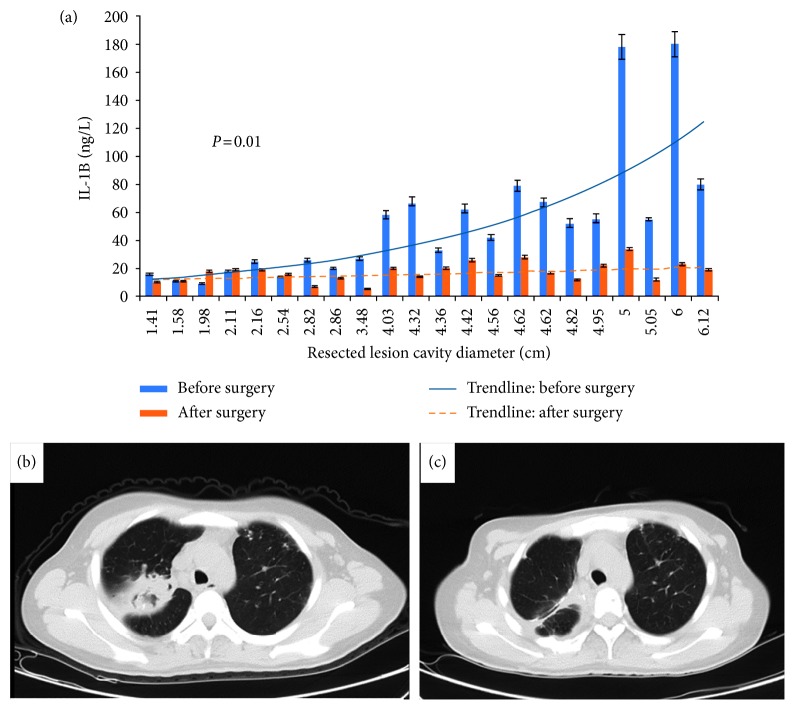
The comparison of IL-1B levels before and after surgery: (a) each dot represents an individual CPA patient. There was a clear upward trend of IL-1B level with the increase of lesion size before the surgery (solid line) and one month later after the surgery; almost all patients' serum IL-1 decreased (61.3 ± 42.7 ng/L vs 23.7 ± 9.4 ng/L, *P*=0.001) (dotted line), especially in patients whose baseline IL-1 levels were over 20 ng/L. (b) The CT scan of a 63-year-old male CPA patient with multiple cavities, pleural thickening, aspergilloma, and consolidation in the left upper lobe. His serum IL-1 was 180 ng/L prior to the surgery. (c) 3 months later after he received a left upper lobectomy, no CPA lesions were seen in his chest CT and his serum IL-1 decreased to 23 ng/L.

**Figure 3:**
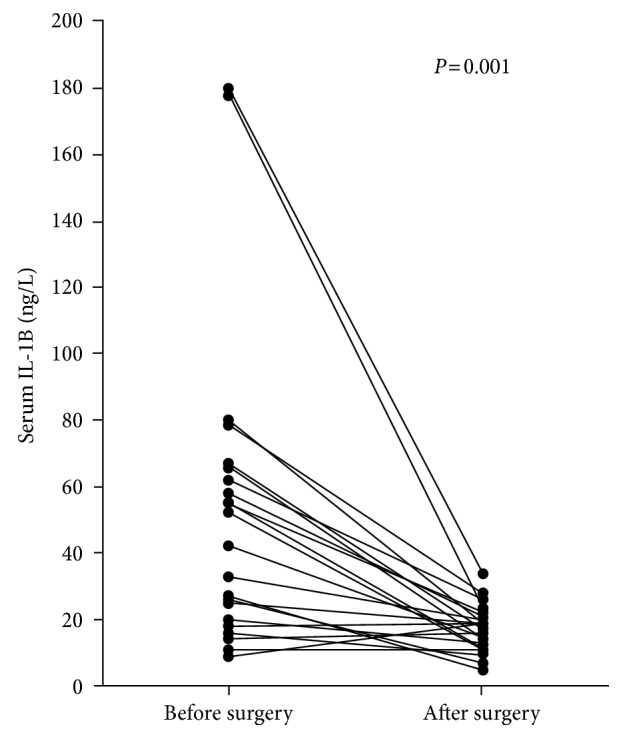
The comparison of serum IL-1B concentrations before and after surgery.

Figure 2 shows the cavity size and the change of serum IL-1B level in 22 patients who got their cavitary lesions surgically resected. There was a clear upward trend of IL-1B with the increase in lesion size before the operation. Three months after surgery, we observed almost universal reduction of serum IL-1B levels (47.4 ± 42.7 ng/L vs 23.7 ± 9.4 ng/L, *P*=0.001) (Figure 3), with most prominent change among those whose baseline IL-1B level was more than 20 ng/L (Figure 2).

## 4. Discussion

To our knowledge, this is the first study exploring correlation between IL-1B level and clinical manifestations in patients with CPA. In our study, CPA patients' underlying disease (mainly TB and COPD), constitutional symptoms (weight loss, productive cough, and hemoptysis), routine laboratory test measures (ESR, CRP, etc.), aspergillus antibody titer, and GM test results were similar to those of reported studies [19]. Moreover, we observed higher serum IL-1B levels in CPA were associated with more and bigger cavitary and aspergilloma lesions, a finding, which were corroborated by a robust reduction in serum IL-1B level after surgical removal of the CPA cavitary lesions. However, this association was substantially confounded by a couple of comorbid conditions and accompanying medications. For example, AECOPD and active TB infection upregulated, while systemic CS or IS therapy suppressed the expression of IL-1B, and thus should be on guard in the clinical use of this cytokine as a monitor of the activity of CPA. The lack in the association between serum IL-1B and cavity wall thickness suggests factors other than IL-1B or inflammatory cytokines play a role.

Although previous studies suggested the associations between CPA and various inflammatory cytokines, e.g., TNF, Tumor growth factor- (TGF-) *β*1, IL-15, and IL-10 [20–23], the results are not consistent and some are difficult to replicate. In 2014, through a comprehensive study containing clinical samples and animal models, Smith et al. revealed a remarkable relationship between IL-1 (and some of its SNPs) and CCPA (main type of CPA) [8]. They speculated that the continued rising of IL-1 might reflect the inflammatory response to the ongoing fungal infection, which could manifest as tissue damage, cavity formation, and aspergilloma formation. Caffrey et al. also confirmed the role of IL-1 in controlling aspergillus fumigatus infection in the murine lung and speculated the important role of IL-1 in pulmonary fungal infection [24]. However, many of these studies are preclinical and focused on the role of IL-1 in pathogenesis rather than using it as a biomarker to trace disease activity. In our study, we have included by far the largest number of CPA cases to study the association of IL-1B level with the disease activity of CPA. We have therefore added another piece of evidence to support the role of IL-1B in CPA, that is, IL-1B is important not only in the pathogenesis of aspergillosis, but also in the activity of this particular disease.

As per the 2016 new guidelines for CPA, the enlarging lung cavitation and/or progression with more tissue damage and likely poor therapeutic responses [16]. Finding a convenient approach to monitor the disease activity is therefore urgently needed. Since the radiographic appearance and histopathological features are closely related to immune and inflammatory response rather than just direct invasion by fungi [1, 25, 26], cytokine should theoretically have the role to monitor CPA activity. This is clinically important since the follow-up evaluation of CPA is still insufficient at this moment. The reported clinical, mycological, or radiological criteria are not standardized and vary from study to study. Although the size or number of cavities and fungus ball, as well as the cavity and pleural wall thickness shown in CT scans are commonly used to assess disease status and treatment response, the criteria are not consistent [17,18,27–29]. In addition, only little change is visible on CT scans 3 months after the inception of antifungal therapy [16]. Due to the requirement of long follow-up intervals, CPA evaluation by chest CT is not the most effective assessment in our clinical work. Other tests such as serum IgG level [30] and GM test [31], etc. have also been considered attractive in monitoring pulmonary fungal infection. However, they are not as good as IL-1B in our study. Since the serum level of IL-1B showed good correlation with CT findings that are indicative of disease activity, it might also be useful to predict therapeutic response for CPA. Indeed, among 22 patients who got their cavitary lesions resected, the IL-1B level was found significantly reduced. We will address whether similar trend can be observed among patients who respond well to antifungal therapy, and how fast the change of IL-1B level will be after treatment in future prospective studies.

Several important issues and limitations should be mentioned here. First, this is a retrospective study conducted only in our hospital; therefore, selection bias is inevitable. Second, our whole group of recruited subjects with CPA is not a homogenous population and it includes patients with a variety of infectious and noninfectious comorbidities and some patients taking systemic CS or IS therapy, which is supposed to affect IL-1B expression. For this problem, our group analyzed the association between IL-1B and disease activity first as a whole and then in a subgroup population without confounding factors affecting IL-1B expression, which were identified by performing a multiple linear regression. And at both levels, our results convincingly support a role for IL-1B in the activity of CPA, especially in the latter. In addition, though IL-1 family contains large numbers of members related to *Aspergillus fumigatus* [32], we only measured IL-1B due to the limitation of the detection kit, which may lead to the missing of some important findings such as factors relating to IL-1B regulator or receptor.

## 5. Conclusions

Nevertheless, our study has shown the association of serum IL-1B level with the disease activity of CPA. The higher level of serum IL-1B is associated with more and bigger cavitary and aspergilloma lesions, which are indicative of more active CPA. In addition, IL-1B level reduced accordingly after lesion resection. Measuring IL-1B level therefore could serve as a convenient way to monitor the activity of CPA and be a potential predictive/prognostic marker for treatment response.

## Figures and Tables

**Table 1:** Clinical characteristics of subjects with CPA in the whole and subgroup analysis.

		Whole group (*n*=469)	Total (*n*=88)	Subgroup		*P* value
				Serum IL-1B ≥ 20.3 ng/L (*n*=44)	Serum IL-1B < 20.3 ng/L (*n*=44)	
Age, years		57 (48.0, 66.0)	55 (42.0, 62.0)	56.5 (46.0, 62.0)	50.5 (40.3, 63.0)	0.356
Gender, male		279 (59.5)	52 (59.1)	24 (54.5)	28 (63.6)	0.386
BMI, kg/m^2^		21.1 ± 3.6	21.4 ± 3.9	21.6 ± 4.1	21.3 ± 3.8	0.729
Smoking history, yes, pulmonary underlying diseases		144 (30.7)	26 (29.5)	11 (25.0)	15 (34.1)	0.350
** **TB	Treated	180 (38.4)	40 (45.5)	22 (50.0)	18 (40.9)	0.392
	Active	87 (18.6)	—	—	—	—
NTM infection		30 (6.4)	—	—	—	—
Other acute respiratory infections		59 (12.6)	—	—	—	—
Lung abscess		12 (2.6)	—	—	—	—
Cancers		60 (12.8)	—	—	—	—
COPD	Stable	212 (45.2)	47 (53.4)	24 (54.5)	23 (52.3)	0.831
	Acute	53 (11.3)	—	—	—	—
Sarcoidosis		34 (7.3)	5 (5.7)	2 (4.5)	3 (6.8)	1.000
AIP		12 (2.6)	—	—	—	—
** **Other ILD		53 (11.3)	4 (4.8)	3 (6.8)	1 (2.3)	0.616
Systemic comorbidities						
** **DM		53 (11.3)	7 (8.0)	3 (6.8)	4 (9.1)	1.000
** **Hypertension		60 (12.8)	14 (15.9)	6 (13.6)	8 (18.2)	0.560
** **Cardiac diseases		60 (12.8)	9 (10.2)	3 (6.8)	6 (13.6)	0.484
Cerebrovascular diseases		14 (2.98)	2 (2.3)	0 (0)	2 (4.5)	0.494
Systemic CS therapy		36 (7.7)	—	—	—	—
Systemic IS therapy		32 (6.8)	—	—	—	—
Pulmonary surgery history		38 (8.1)	9 (10.2)	2 (4.5)	7 (15.9)	0.157
Symptoms						
** **Cough		460 (98.1)	85 (96.6)	44 (100)	41 (93.2)	0.241
** **Sputum production		455 (97.0)	82 (93.2)	42 (95.5)	40 (90.9)	0.676
** **Hemosputum		272 (58.0)	45 (51.1)	26 (59.1)	19 (43.2)	0.135
** **Fever		158 (33.7)	19 (21.6)	8 (18.2)	11 (25.0)	0.437
** **Chest pain		58 (12.36)	5 (5.68)	2 (4.5)	3 (6.8)	1.000
** **Fatigue		79 (16.8)	4 (4.5)	3 (6.8)	1 (2.3)	0.616
Radiological features						
** **Cavity		235 (50.1)	40 (45.5)	27 (61.4)	13 (29.5)	0.003
** **Aspergilloma		212 (45.2)	36 (40.9)	25 (56.8)	11 (25.0)	0.002
** **Bronchiectasis		408 (87.0)	83 (94.3)	23 (52.3)	23 (52.3)	1.000
** **Patch		451 (96.2)	82 (93.2)	43 (97.7)	39 (88.6)	0.091
** **Consolidation		363 (77.4)	60 (68.2)	27 (61.4)	33 (75.0)	0.170
** **Nodules		332 (70.8)	58 (65.9)	31 (70.5)	27 (61.4)	0.368
** **Tree-in-bud pattern		85 (18.1)	8 (9.1)	3 (6.8)	5 (11.4)	0.713
** **Atelectasis		28 (6.0)	3 (3.40)	0 (0)	3 (6.8)	0.241
** **Pleural effusion		79 (16.8)	8 (9.1)	5 (11.4)	3 (6.8)	0.713
** **Pleural thickening		192 (40.9)	39 (44.3)	22 (50)	17 (38.6)	0.283
** **Interstitial fibrosis		53 (11.3)	4 (4.8)	2 (4.5)	2 (4.5)	1.000
** **Lung volume reduction		47 (10.0)	6 (6.8)	2 (4.5)	4 (9.1)	0.676
** **GGO		53 (11.3)	8 (9.1)	2 (4.5)	6 (13.6)	0.266
** **Cavity diameter (cm)		3.6 (2.6, 4.8)	3.4 (2.1, 4.8)	4.6 (2.8, 5.0)	2.0 (1.6, 2.7)	0.000
** **Aspergilloma size (cm)		2.4 (1.7, 3.3)	1.8 (1.5, 3.5)	2.5 (1.6, 3.8)	1.5 (0.9, 1.9)	0.006
** **Cavity wall thickness (cm)		0.7 (0.5, 1.0)	0.7 (0.3, 1.1)	0.9 (0.4, 1.2)	0.4 (0.2, 0.7)	0.023
G test, positive		95 (20.3)	16 (18.2)	9 (20.5)	7 (15.9)	0.580
GM test, positive						
** **Blood		211 (45.0)	43 (48.9)	25 (56.8)	18 (40.9)	0.135
** **BWF		338 (72.1)	60 (68.2)	32 (72.7)	28 (63.6)	0.360
Aspergillus antibody, positive		276 (58.8)	55 (62.5)	25 (56.8)	30 (68.2)	0.271
Culture proof						
** **Sputum		177 (37.7)	32 (36.4)	17 (38.6)	15 (34.1)	0.658
** **BWF		165 (35.2)	32 (36.4)	19 (43.2)	13 (29.5)	0.184
Laboratory test result						
** **ALB		39.1 ± 5.7	38.9 ± 5.6	38.6 ± 4.9	39.1 ± 6.2	0.699
** **WBC		7.2 (5.4, 9.3)	7.5 (5.3, 9.0)	7.1 (5.1, 9.6)	8.0 (5.4, 8.8)	0.867
** **CRP		6.9 (2.7, 20.4)	5.9 (2.1, 18.8)	5.8 (2.0, 26.2)	6.0 (2.4, 8.3)	0.987
** **ESR		33.3 (16.4, 51.3)	26.9 (11.8, 51.6)	26.3 (11.5, 50.0)	30.7 (12.2, 52.7)	0.739
Inflammatory cytokines (ng/l)						
** **IL-1B		26.1 (15.2, 50.6)	20.3 (15.2, 42.3)	42.2 (25.2, 57.6)	15.2 (11.7, 17.3)	0.000
** **IL-2		144.9 (102.2, 193.1)	110.9 (75.1, 159.8)	112.5 (76.3, 161.1)	101.0 (69.9, 147.3)	0.413
** **IL-5		39.2 (26.8, 62.3)	37.7 (26.4, 61.5)	46.8 (27.0, 62.4)	32.8 (25.1, 50.1)	0.090
** **IL-6		45.6 (23.0, 94.8)	39.8 (26.7, 73.9)	56.0 (27.6, 77.2)	33.9 (24.6, 66.6)	0.123
** **TNF-*α*		52.3 (30.3, 96.1)	53.6 (33.7, 90.0)	65.4 (33.5, 96.0)	46.5 (33.9, 79.4)	0.174
** ** *γ*-IFN		16.6 (11.6, 27.5)	17.8 (11.8, 27.0)	20.0 (11.9, 31.4)	16.8 (11.7, 22.3)	0.129

Data are presented as mean ± standard deviation or median (interquartile range) or number (percentage). AIP: acute interstitial pneumonia; ALB: albumin; BMI: body mass index; BWF: bronchial washing fluid; COPD: chronic obstructive pulmonary disease; CRP: C-reactive protein; CS: corticosteroid; DM: diabetes mellitus; ESR: erythrocyte sedimentation rate; GGO: ground glass opacity; IFN: interferon; IL: interleukin; ILD: interstitial lung disease; IS: immunosuppressive; NTM: nontuberculous mycobacterium; TB: tuberculosis; TNF: tumor necrosis factor; WBC: white blood cell.

**Table 2:** Multiple linear regression model for predicting serum IL-1B using age, gender, BMI, smoking status, TB, NTM infection, lung abscess, other acute respiratory infections, cancer, COPD, AIP, sarcoidosis, other interstitial diseases, diabetes mellitus, hypertension, cardiac disease, cerebrovascular disease, systemic CS or IS therapy, and pulmonary surgery history as covariates in all the subjects with chronic pulmonary aspergillosis.

Comorbid conditions		*β* Coefficient	Standard error	*P* value
TB	None (reference)	—	—	—
	Treated	0.003	0.106	0.978
	Active/being treated	0.475	0.138	0.001
NTM infection		0.538	0.196	0.006
Lung abscess		0.975	0.304	0.001
Other acute respiratory infection		0.547	0.151	0.000
Cancer		0.542	0.147	0.000
COPD	None (reference)	—	—	—
	Stable	0.119	0.102	0.247
	Acute	0.612	0.164	0.000
AIP		0.818	0.305	0.008
Sarcoidosis		0.407	0.185	0.028
Systemic CS or IS therapy		−0.297	0.144	0.039

AIP: acute interstitial pneumonia; COPD: chronic obstructive pulmonary disease; CS: corticosteroid; ILD: interstitial lung disease; IS: immunosuppressive; NTM: nontuberculous mycobacterium.

**Table 3:** Correlation of serum inflammatory factors with the activity of chronic pulmonary aspergillosis in the whole subjects.

	Cavity diameter		Aspergilloma size		Cavity wall thickness	
	Coefficient	*P* value	Coefficient	*P* value	Coefficient	*P* value
WBC	0.088	0.149	0.034	0.587	0.065	0.282
ESR	0.075	0.215	0.097	0.118	0.098	0.109
CRP	0.113	0.064	0.078	0.210	0.103	0.089
IL-1B	0.128	0.035	0.123	0.047	0.043	0.479
IL-2	0.073	0.229	0.014	0.827	0.094	0.122
IL-5	0.053	0.387	0.098	0.116	0.085	0.160
IL-6	0.091	0.135	0.079	0.207	0.084	0.168
TNF-*α*	0.083	0.174	0.049	0.435	0.094	0.121
*γ*-IFN	0.063	0.300	0.060	0.337	0.052	0.396

CRP: C-reactive protein; ESR: erythrocyte sedimentation rate; IFN: interferon; IL: interleukin; TNF: tumor necrosis factor; WBC: white blood cell.

## Data Availability

The data used to support the findings of this study are included within the article.
